# Supplementary figures and images for: 3D-Fast Gray Matter Acquisition with Phase Sensitive Inversion Recovery Magnetic Resonance Imaging at 3 Tesla: Application for detection of spinal cord lesions in patients with multiple sclerosis
Source: PLoS One. 2021 Apr 22;16(4):e0247813. doi: 10.1371/journal.pone.0247813 (PMC8061976; doi:10.1371/journal.pone.0247813)

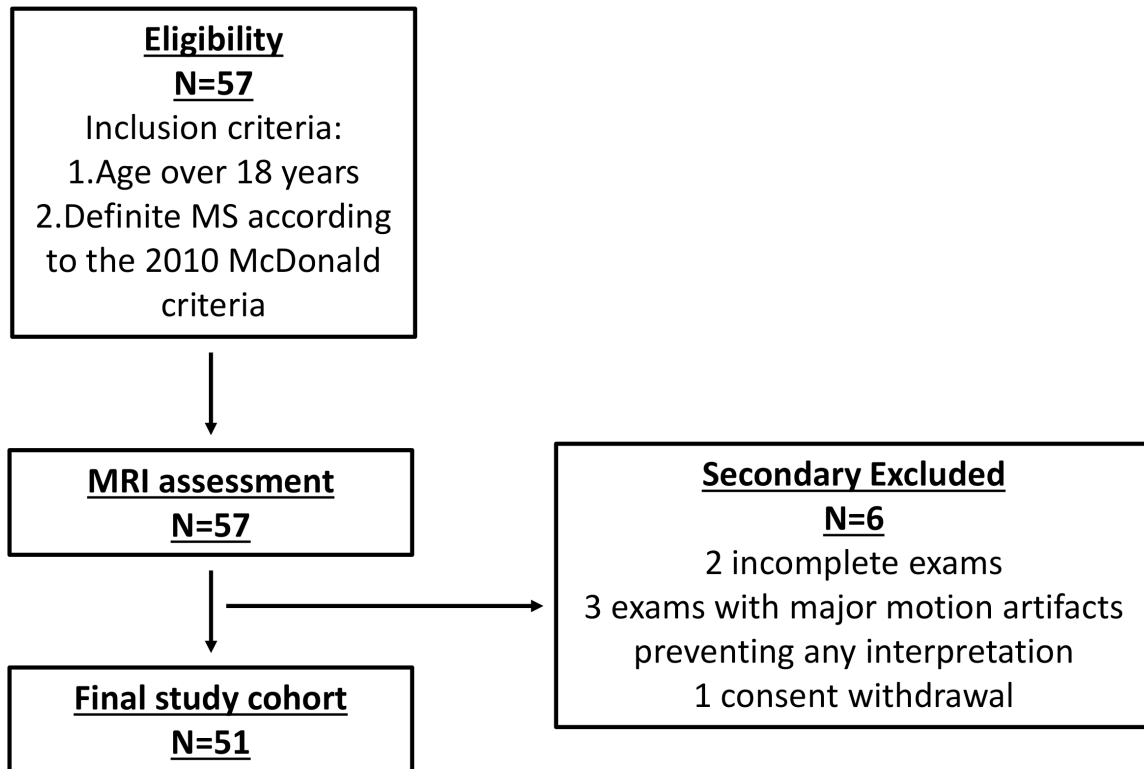

Supplementary Figure 1

Supplement: S1 Fig — MS: Multiple Sclerosis; MRI: Magnetic Resonance Imaging. (PDF) [file pone.0247813.s001.pdf]

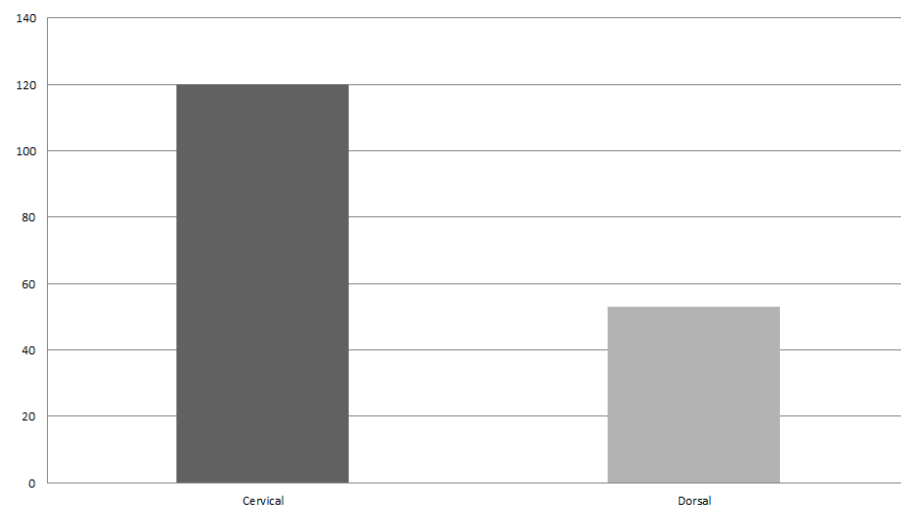

Supplementary Figure 2.a

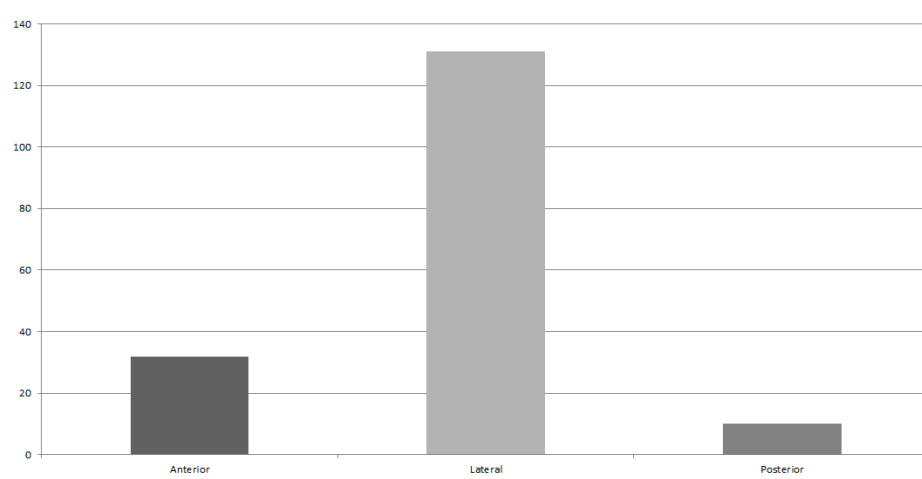

Supplementary Figure 2.b

Supplement: S2 Fig — y-axis indicate the number of lesions. (PDF) [file pone.0247813.s002.pdf]
