# Supplementary material for: 3D-Fast Gray Matter Acquisition with Phase Sensitive Inversion Recovery Magnetic Resonance Imaging at 3 Tesla: Application for detection of spinal cord lesions in patients with multiple sclerosis
Source: PLoS One. 2021 Apr 22;16(4):e0247813. doi: 10.1371/journal.pone.0247813 (PMC8061976; doi:10.1371/journal.pone.0247813)
Supplement: S1 Table — sd: standard deviation; IQR: Interquartile ratio; EDSS: Expanded Disability Status Scale. (DOCX) [file pone.0247813.s003.docx]

|  | | Secondary excluded patients (n=6 patients) | Final study cohort (n=51 patients) | *p* |
| --- | --- | --- | --- | --- |
| Gender | Men | 2 (33%) | 17 (33%) | *0.9* |
|  | Women | 4 (67%) | 34 (67%) |  |
| Age (years)(mean [sd]) | | 44 [23.1] | 43 [22.5] | *1* |
| EDSS (median [IQR]) | | 3 [3.4] | 3 [3.5] | *1* |
| Disease Duration (years)(median [IQR]) | | 7.2 [9.9] | 7.5 [10.5] | *0.9* |

## Supplementary Table 1

Comparison between secondary excluded patients and patients enrolled in the final study cohort. sd: standard deviation; IQR: Interquartile ratio; EDSS: Expanded Disability Status Scale
