# Supplementary material for: 3D-Fast Gray Matter Acquisition with Phase Sensitive Inversion Recovery Magnetic Resonance Imaging at 3 Tesla: Application for detection of spinal cord lesions in patients with multiple sclerosis
Source: PLoS One. 2021 Apr 22;16(4):e0247813. doi: 10.1371/journal.pone.0247813 (PMC8061976; doi:10.1371/journal.pone.0247813)
Supplement: S2 Table — (DOCX) [file pone.0247813.s004.docx]

|  |  |  | Relapsing-Remitting MS (n=38 patients) | Secondary-Progressive MS (n=8 patients) | Primary-Progressive MS (n=5 patients) | Overall |
| --- | --- | --- | --- | --- | --- | --- |
| Sagittal plane | Cervical level | 3D-FGAPSIR | 159 | 33 | 22 | 214 |
|  |  | Conventional dataset | 70 | 15 | 9 | 94 |
|  | Dorsal level | 3D-FGAPSIR | 97 | 20 | 13 | 130 |
|  |  | Conventional dataset | 57 | 12 | 8 | 77 |
| Axial plane | Anterior | 3D-FGAPSIR | 28 | 6 | 4 | 38 |
|  |  | Conventional dataset | 3 | 1 | 1 | 5 |
|  | Posterior | 3D-FGAPSIR | 95 | 20 | 13 | 128 |
|  |  | Conventional dataset | 89 | 16 | 14 | 119 |
|  | Lateral | 3D-FGAPSIR | 132 | 28 | 28 | 178 |
|  |  | Conventional dataset | 35 | 7 | 5 | 47 |

## Supplementary Table 2

Detailed locations of spinal cord lesions according to the type of Multiple Sclerosis (MS).
