# Supplementary material for: 3D-Fast Gray Matter Acquisition with Phase Sensitive Inversion Recovery Magnetic Resonance Imaging at 3 Tesla: Application for detection of spinal cord lesions in patients with multiple sclerosis
Source: PLoS One. 2021 Apr 22;16(4):e0247813. doi: 10.1371/journal.pone.0247813 (PMC8061976; doi:10.1371/journal.pone.0247813)
Supplement: Attachment — Submitted filename: Response to Reviewer.docx [file pone.0247813.s005.docx]

Reviewer #1:

**R1.1 : In this work the authors propose the use of a new fast 3D PSIR sequence for the detection of multiple sclerosis (MS) lesions in the spinal cord. The application of such a sequence in the brain and also in the spinal cord has been already shown to be effective for the detection of MS lesions. In particular, in the spinal cord, this sequence has been suggested also for the segmentation of the grey matter tissue. Authors starts from these same evidences to motivate the application of this sequence in MS with some innovations. However, although the title suggests some technical innovations, these are not well introduced and explained. The details of the changes are referred to other two papers, but it remains unclear what is the original idea of the present work. If the novelty is the adjustment of the inversion time to null the signal from the white matter, as done in reference 13 for an MPRAGE sequence, this should be better clarified and the reason why this should improve contrast between lesions and the surrounding tissue proven.**

We thank the Reviewer for his/her comment. We agree that the technical innovations associated with the FGAPSIR sequence were not sufficiently well explained. Both the introduction and methods have been rephrased to clarify the modifications performed on the FGAPSIR sequence, as follows:

1) Introduction

“Our center developed a 3T MRI high resolution sequence which aims to improve the lesion to normal cord contrast.”

2) Methods

“The 3D-FGAPSIR was developed combining a Fast Grey Matter Acquisition T1 Inversion Recovery (FGATIR, 13) and a 3D-PSIR sequence optimized for spinal cord visualization (11). Starting from the 3D-PSIR sequence, we modified the Turbo Field Echo inversion prepulse and the shot duration in order to increase the possible T1 contrast range and to obtain a Fast Grey Matter Acquisition T1 Inversion Recovery contrast on the magnitude image. The shot duration was set at 400ms, as performed for the FGATIR (13), which improves the lesion to white matter contrast, due to the longer T1 relaxation of MS lesions compared to white matter (Lommers et al,2019).”

*Lommers, E., Simon, J., Reuter, G., Delrue, G., Dive, D., Degueldre, C., ... & Maquet, P. (2019). Multiparameter MRI quantification of microstructural tissue alterations in multiple sclerosis. NeuroImage: Clinical, 23, 101879.* *doi: 10.1016/j.nicl.2019.101879*

**In fact, it is not obvious that zeroing the signal from the white matter will increase the contrast with lesions. Especially for a paper suggesting technical advances, the inversion time should be optimized to this aim analytically, for instance, and the new solution validated by comparing the contrast with the original PSIR, to demonstrate that this solution is effective.**

The shot duration was set at 400ms following the parameters of the cited papers (Fechner et al, 2019; Sudhyadhom et al, 2009), which have already demonstrated an improvement of the lesion to white matter contrast, as well as increased detection of MS lesions (Fechner et al, 2019). However, we agree with the Reviewer that analytical optimization could be performed to improve the lesion to white matter contrast. This optimization could be performed following the signal equation of an IR sequence and an inversion time chosen to null the signal intensity amplitude of the white matter:

$$S_{IR}\left( T1,TE,TR \right)=\rho(1-2e^{-\frac{TI}{T1}}+2e^{-\frac{\left( TR-\frac{TE}{2} \right)}{T1}}-e^{-\frac{TR}{T1}})e^{-\frac{TE}{T2}}$$

*Hou, P., Hasan, K. M., Sitton, C. W., Wolinsky, J. S., & Narayana, P. A. (2005). Phase-sensitive T1 inversion recovery imaging: a time-efficient interleaved technique for improved tissue contrast in neuroimaging. American journal of Neuroradiology, 26(6), 1432-1438.*

Using our TR and TE, the inversion time might be optimal between 500-600ms to allow an efficient suppression of the white matter (figure below), and thus an increase of the lesion to white matter contrast. This optimization needs more investigation, and the sequences parameters might be optimized in the following studies.

We agree that a direct comparison with the original PSIR is relevant. However, our study was a preliminary study whose aim was to validate the FGAPSIR as compared to a conventional imaging dataset. Further studies will be conducted to compare the FGAPSIR and the original PSIR sequences.

We highlighted these points in the Limitations section of our manuscript.

**It should also be considered that when the TI is set to nulled the white matter all the other relevant tissues, having a longer T1, are acquired when the magnetization is still along the negative y axis and the expanded contrast of a phase contrast image preserved; so I am wondering about the need of obtaining a reference scan, as done in this PSIR sequence, for correcting the background phase variations.**

Indeed, the FGAPSIR phase-corrected image was calculated using a reference scan, as done in the PSIR sequence. We highlighted this point in the Material and Methods section.

**R1.2 Another point is about the comparison with the 3D STIR sequence, similar to the proposed sequence for resolution, but also in the way the contrast is constructed: the discrepancy between the number of lesions identified is really high.
All these aspects should be better considered in the discussion of the paper from a technical point of view.**

We agree that the discrepancy between the number of lesions identified with the 3D STIR and the 3D FGAPSIR is high. As you mentioned, the 3D STIR sequence we performed in our study matched the 3D FGAPSIR for resolution and met the technical requirements recommended by the international guidelines. Moreover, our results are in line with a recently published study comparing a 3D PSIR sequence with a 3D STIR (Mirafzal et al 2020), showing more than twice as many lesions with the 3D PSIR sequence. We highlighted this point in the discussion section.

*Mirafzal S, Goujon A, Deschamps R, Zuber K, Sadik JC, Gout O, Lecler A, Savatovsky J. 3D PSIR MRI at 3 Tesla improves detection of spinal cord lesions in multiple sclerosis. J Neurol. 2020 Feb;267(2):406-414. doi: 10.1007/s00415-019-09591-8. Epub 2019 Oct 26.)*

**R1.3. Overall I found the discussion too optimistic about the potentiality of the sequence and I found some conclusions, such that of suggesting its use for the atrophy measurements or that this single sequence could replace the whole recommended protocol for the spinal cord in MS, too simplistic.**

We agree that our study was a preliminary study and that the discussion might be too optimistic. As suggested, we deleted the sentences not directly supported by the results of our study.

These are other additional comments:
**R1.4 In the introduction it is stated that “the sensitivity of conventional imaging like T2-weighted imaging to show spinal cord lesions is low” This sentence simplifies the concept without giving details. Could authors elaborate a little more on this?**

As requested, we elaborated more on this statement in the introduction section.

**R1.5 “Optimized MR sequences have been developed and show significant improvement of detection and delineation of lesions” Following point 1, could authors explain the major improvements introduced in these non- conventional techniques?**
We added a sentence in the introduction section to explain the major improvements introduced in these non- conventional techniques.

**R 1.6 One of the exclusion criteria was the insufficient quality of MRI for the interpretation, due to the presence of artifacts. However the robustness against artifacts should be an important characteristic to be reported and analyses. Authors should describe these cases instead.**
We agree the robustness against artifacts is an important characteristic. Therefore, we provided data regarding the presence of artifacts in the Results section. We added a sentence in the Material and Methods section accordingly.

**R1.7 MRI protocol. It seems that all sequences were acquired after contrast injection. The international guidelines prescribe a pre-contrast protocol followed by a post contrast T1 weighted sequence, in case there are T2-hyper-intense visible lesions. Please explain your choice of acquiring all sequences after contrast injection. Furthermore I would add the direct reference for the recommended MR protocol in MS, since the cited paper refers to other papers about this point.**

All sequences were acquired after contrast injection, which is the protocol for all our patients with MS. The occurrence of a pre contrast T1 hyper intense lesion is exceedingly rare, and our protocol is designed to increase the delay between gadolinium injection and completion of the MRI sequences, which allows for better detection of MS lesions. This protocol follows international guidelines.

As requested, we added the direct references for recommended MR protocols in MS.

**R1.8. Sequence parameters: for the PSIR sequence the flip angle for the reference image is usually shorter than that of the inversion recovery part of the sequence. If this is the case, the second flip angle should be specified. For the 3D STIR sequence, was a variable flip angle used to compensate for the long echo train required to keep acquisition time short enough? Could this have affected the quality of the images, because of the induced filtering effect?**
As requested we specified the second flip angle in Table 1. The flip angle for the reference image is shorter than that of the inversion recovery part of the sequence.

Regarding the 3D STIR sequence, the flip angle was kept constant to obtain the best quality image possible.

**R1.9. In the discussion it is stated that characteristic of the sequence is the increased contrast of spinal cord lesions by combining the benefits of nulling normal white matter, suppression of surrounding CSF signals. This is not totally true or at least this is not necessarily the optimal condition for the contrast. Also, in this sequence, CSF is not nulled, but the signal appears dark because of the negative values.**

We agree this statement was imprecise. We modified the discussion accordingly.

Reviewer #2: Summary
The authors present prospective single-center data that convincingly demonstrate spinal cord lesion detection via 3D-Fast Gray Matter Acquisition with Phase Sensitive Inversion Recovery (3D-FGAPSIR) is improved as compared to conventional 3D-Short-Tau Inversion Recovery (3D-STIR) and sagittal T1- and T2-weighted sequences at 3 Tesla in 51 subjects with MS. 3D-FGAPSIR detected more lesions than the conventional methods and offered improved lesion delineation and lesion-to-cord contrast ratio. In general, the study is well designed, the observations are novel and clinically relevant. I think the study could be published with some minor revisions.

Comments by Section
**R2.1 Methods, Participants, Page 6: Did the 6 omitted subjects differ from the larger group in any meaningful way (age, sex, disease category, MRI features)? This could be added to the Supplementary Data.**

There were no significant differences between the 6 omitted subjects and the final study cohort. We provided Supplementary Table 1 to report these results.

**R2.2 Methods, MRI Analysis, Page 8-9: Have similar lesion delineation and reader-reported confidence scales been used in prior work? Would cite if so.**

To the best of our knowledge, similar lesion delineation and reader-reported confidence scales have not been used in prior work.

**R2.3 Results, Lesion Detection Page 11: Did lesion detection via 3D-FGAPSIR outperform conventional imaging differentially based on MS disease stage. For example, with longstanding disease cord lesions made become less apparent over time. Some kind of analysis of this breakdown might be a useful addition. As a suggestion, the lesion data (cervical/dorsal, anterior/lateral) presented in the results here might also be shown in a separate table similar to table 2, comparing RRMS, SPMS, PPMS and Overall.**

We agree that this analysis is valuable. As we enrolled a majority of patients with RRMS and only 13 patients with progressive stages in this prospective study, our statistician was unable to conduct statistic studies on such small samples. Therefore, we provided only raw data regarding the number of spinal cord lesions detected by each dataset according to the type of MS in Supplementary Table 2.

**R2.4. Results, Lesion Detection Page 11: It would be interesting to report the details of the lesions seen by 3D-FGAPSIR that conventional imaging missed. Consider showing this in a Figure.**

As requested, we provided Supplementary Figure 2 to report the details of these missed lesions.

**R2.5. Results, “Correlation with clinical data”: Analysis of relationship to clinical status could be strengthened by other outcomes that may better reflect damage to the cord. Analysis of association to ambulatory walk time if performed, EDSS subscores (sensorimotor function, bowel bladder function vs. cerebral, cerebellar, brainstem), for example might be means of expanding that clinical analysis. I appreciate that this data may not be available and such analysis beyond the scope of this imaging study.**

We agree that such analysis might be valuable. Unfortunately, we collected only EDSS when enrolling patients in our prospective study, thus this data is not available. However, we intend to launch a more comprehensive validation study including detailed clinical scores and scales to look for clinico-radiological correlations.

**R2.6. Results, “Correlation with clinical data”: Similar to prior comment, EDSS linkage to lesions could be assessed for RRMS vs. progressive stages perhaps to shed light on 3D-FGAPSIR clinical utility.**

There was an overall better correlation between the severity of EDSS scores and the number of lesions detected on 3D-FGAPSIR in progressive stages versus in RRMS patients: 0.9 versus 0.4. However, as mentioned before, we enrolled a majority of patients with RRMS and only 13 patients with progressive stages in this prospective study, limiting our ability to conduct statistical studies on such small samples. As recommended by our statistician, we indicated raw correlation values only without associating a statistical test to them. We modified the Results section and the discussion section.

**R2.7. Discussion, Line 5: The Discussion could expand upon how the improvement in lesion detection by FGAPSIR over conventional methods compares to other similar technical advances noted in the studies cited here.**

As requested, we modified the discussion to elaborate on that point.

**R2.8. Tables & Figures: No major concerns. My only comment would be that panels/images of the axial reconstructed view may helpful to demonstrate the author’s assertion regarding the superiority of FGAPSIR over conventional techniques for anterior and lateral locations.**

We added axial reconstructed views in the Figure 1, in addition to the ones displayed in Figure 2, to highlight the superiority of FGAPSIR over conventional techniques.

Minor Comments/Grammar Suggestions

**R2.9. Introduction, Page 5, Line 6: Minor grammatical edit. Would suggest use of the phrase “can be challenging” rather than “remains a challenge” and delete “to” in the phrase “to image artifacts”.**

We modified the sentence.

**R2.10. Introduction, Page 5 Line 10: I would clarify “spinal cord” lesion burden and “in MS”.**

As requested, we clarified the sentence.

**R2.11. Introduction, Page 5 Line 13-14: Delete the word “showing” here, change phrasing to “active enhancement in the cord”.**

As requested, we modified the sentence.

**R2.12. Introduction, Page 5 Line 16: Use “performance” (singular). This is duplicated in the Discussion, Page 13, Line 15.**

We corrected this error.

**R2.13. Introduction, Page 5 Line 19: The aim was to “evaluate the detection of spinal cord lesions by 3D-FGAPSIR as compared to…”**

As requested we modified the sentence.

**R2.14. Methods, Page 7, MRI Analysis. Line 3: “Height” (spelling).**

We corrected this typo.

**R2.15. Methods, Page 7, MRI Analysis. Line 6: “neuroradiologist” (singular).**

We corrected this typo.

**R2.16. Methods, Page 7, MRI Analysis. Line 7: Delete the word “later”.**
As requested we deleted the word later.

**R2.17. Methods, Page 8, MRI Analysis. Line 4: positive and negative predictive value need not be capitalized. This is repeated in the final paragraph of the Discussion as well.**

We modified the sentences in both methods and the discussion sections.

**R2.18. Methods, Page 8, MRI Analysis. Line 17-18: Correct the spacing “locationonto” and would delete word “most” from “most predominant”.**

We corrected the sentence.

**R2.19. Results, Lesion Detection: The subtitle should be capitalized consistent, so “Lesion detection".**

We corrected this inconsistency.

**R2.20. Discussion, Page 14, Line 19: Change phrasing to “3D-FGAPSIR might also improve the assessment of disability”.**

As requested, we modified the sentence.

**R2.21. Discussion, Page 15, Line 6: patients who “may be less likely to hold still”.**

As requested, we modified the sentence.
