# Supplementary material for: 3D-Fast Gray Matter Acquisition with Phase Sensitive Inversion Recovery Magnetic Resonance Imaging at 3 Tesla: Application for detection of spinal cord lesions in patients with multiple sclerosis
Source: PLoS One. 2021 Apr 22;16(4):e0247813. doi: 10.1371/journal.pone.0247813 (PMC8061976; doi:10.1371/journal.pone.0247813)
Supplement: Attachment — Submitted filename: Response to Reviewer V3.docx [file pone.0247813.s006.docx]

**Review Comments to the Author :**

Reviewer #1: Although authors tried to address my concerns, the answers provided and the changes made to the manuscript do not sufficiently clarify the points of my questions. It is still not evident the key idea for the technical improvement presented, that the contrast is optimally maximized with this solution and the need of applying the phase sensitization in this particular case, when the magnetization of the relevant tissues is still along the negative axis. The new reference included (Mirafzal et al. J Neurol. 2020) has already shown the advantage of using the 3DPSIR sequence against a 3D STIR sequence; so what is missing is the advantage of using a PSIR sequence with the proposed inversion time, optimized to suppress the WM, versus the conventional 3D PSIR.
**Overall I am satisfied with the answers to my questions, except for questions 1 and 5, that should be reconsidered.**

Reviewer #2: The authors have submitted a revision that addresses reviewer suggestions and strengthens the manuscript. suggestions from the initial critique could not be addressed due to statistical power or study design, and the authors discuss these limitations. The manuscript should be published. The following additions offer improvement on the initial submission:
- Newly added Supplementary Table 1 compares features of included/excluded subjects.
- Supplementary Table 2 updated to show disease stage.
- Supplementary Figure 2 shows examples case of lesions seen by 3D-FGAPSIR that conventional imaging missed.
- The Discussion was modified in response to the point related to disease stage correlation between the severity of EDSS scores and the number of lesions detected on 3D-FGAPSIR. The authors found an overall better correlation between the severity of EDSS scores and the number of lesions detected on 3D-FGAPSIR in progressive stages versus in RRMS patients: 0.9 versus 0.4 though opt to present only raw data given that the subgroup analysis is underpowered (13 patients with progressive MS)
(R2.6. Results, “Correlation with clinical data”: Similar to prior comment, EDSS linkage to lesions could be assessed for RRMS vs. progressive stages perhaps to shed light on 3D-FGAPSIR clinical utility.)
- The Discussion was expanded in a thoughtful way that addresses the critique, noting how lesion detection by FGAPSIR offers improvement to conventional and similar alternative methods.
- Additional axial reconstructed images strengthen the case examples illustrated in Figure 1.
- All minor grammatical issues have been corrected.

Reviewer #1: Questions 1 & 5:

**Question 1: In this work the authors propose the use of a new fast 3D PSIR sequence for the detection of multiple sclerosis (MS) lesions in the spinal cord. The application of such a sequence in the brain and also in the spinal cord has been already shown to be effective for the detection of MS lesions. In particular, in the spinal cord, this sequence has been suggested also for the segmentation of the grey matter tissue. Authors starts from these same evidences to motivate the application of this sequence in MS with some innovations. However, although the title suggests some technical innovations, these are not well introduced and explained. The details of the changes are referred to other two papers, but it remains unclear what is the original idea of the present work. If the novelty is the adjustment of the inversion time to null the signal from the white matter, as done in reference 13 for an MPRAGE sequence, this should be better clarified and the reason why this should improve contrast between lesions and the surrounding tissue proven.**

**R1.1** The advantage of the 3D FGAPSIR is that the range of the T1-weighted is increased, ranging from -1 to 1 instead of 0 to 1 in a T1 SE sequence. This provides improved grey matter-to-white matter contrast with clear lesion delineation as well as hypointense CSF signal intensity because of its large negative magnetization. This offers a good visualization of spinal cord lesions which appear hypointense in the white matter of the spinal cord. We developed the Material and Methods section to explain more in details the technical advantages of the 3D FGAPSIR sequence.

In the proposed sequence, the signal was not optimally maximized as the TI was not perfectly set to suppress the WM. This part needs prospective study to properly set the inversion. Moreover, we totally agree with the Reviewer, that a study investigating the advantage of using the proposed PSIR sequence versus the conventional 3D PSIR is needed. We mentioned these limitations in the limitation section.

**Question 2: “Optimized MR sequences have been developed and show significant improvement of detection and delineation of lesions” Following point 1, could authors explain the major improvements introduced in these non- conventional techniques?**
**R1.5** We modified and expanded the introduction section to explain more in details the major improvements introduced in these non- conventional techniques, from both technical and clinical points of view.
