# Supplementary material for: 3D-Fast Gray Matter Acquisition with Phase Sensitive Inversion Recovery Magnetic Resonance Imaging at 3 Tesla: Application for detection of spinal cord lesions in patients with multiple sclerosis
Source: PLoS One. 2021 Apr 22;16(4):e0247813. doi: 10.1371/journal.pone.0247813 (PMC8061976; doi:10.1371/journal.pone.0247813)
Supplement: Attachment — Submitted filename: Response to Reviewer V4.docx [file pone.0247813.s007.docx]

**Review Comments to the Author :**

Reviewer #1: I appreciate the changes made and the attempt of better explaining the technical aspects. However these are still insufficient in my opinion for a technical paper. My suggestion is to remove the sentences that emphasize technical improvements and innovations.
**1.1** For instance the title could be changed in this way:
"3D-Fast Gray Matter Acquisition with Phase Sensitive Inversion Recovery Magnetic Resonance Imaging at 3 Tesla: application for detection of spinal cord lesions in patients with multiple sclerosis"

**R1.1 The title was changed as requested.**

**1.2** At the end of the introduction I would change the sentence "Our center developed a 3T MRI high resolution sequence" in something like "We optimised the inversion time of an MPRAGE sequence to null the white matter …

**R1.2: As suggested, we have modified this sentence to “*We optimized the inversion time of a 3D PSIR sequence to null the white matter signal, which aims to improve the lesion to normal cord contrast.*”**

**1.3** I would recommend not to mention that the contrast between lesions and the background was optimised, because this is not. This is a finding, not an aim of the study. I am referring to the sentence in the discussion when Authors write "3D-FGAPSIR was specifically targeted to increase the contrast of spinal cord lesions by combining ..."
In the same way the other similar sentence along the manuscript.

**R1.3: As suggested, we deleted the sentence mentioning the optimization of the contrast between lesions and the background in the discussion.**
